# Constitutive expression of transgenes encoding derivatives of the synthetic antimicrobial peptide BP100: impact on rice host plant fitness

**DOI:** 10.1186/1471-2229-12-159

**Published:** 2012-09-04

**Authors:** Anna Nadal, Maria Montero, Nuri Company, Esther Badosa, Joaquima Messeguer, Laura Montesinos, Emilio Montesinos, Maria Pla

**Affiliations:** 1 Institute of Food and Agricultural Technology (INTEA), University of Girona, Campus Montilivi, EPS-1 17071, Girona, Spain; 2 Plant Genetics Department, Centre de Recerca en Agrigenòmica CSIC-IRTA-UAB-UB, Carretera de Cabrils, Km 2, 08348, Barcelona, Spain

**Keywords:** Antimicrobial peptide AMP, BP100, Transgenic rice, Oryza sativa, Hostplant fitness, Pathogen-resistant rice

## Abstract

**Background:**

The Biopeptide BP100 is a synthetic and strongly cationic α-helical undecapeptide with high, specific antibacterial activity against economically important plant-pathogenic bacteria, and very low toxicity. It was selected from a library of synthetic peptides, along with other peptides with activities against relevant bacterial and fungal species. Expression of the BP100 series of peptides in plants is of major interest to establish disease-resistant plants and facilitate molecular farming. Specific challenges were the small length, peptide degradation by plant proteases and toxicity to the host plant. Here we approached the expression of the BP100 peptide series in plants using BP100 as a proof-of-concept.

**Results:**

Our design considered up to three tandemly arranged BP100 units and peptide accumulation in the endoplasmic reticulum (ER), analyzing five BP100 derivatives. The ER retention sequence did not reduce the antimicrobial activity of chemically synthesized BP100 derivatives, making this strategy possible. Transformation with sequences encoding BP100 derivatives (*bp100der*) was over ten-fold less efficient than that of the hygromycin phosphotransferase (*hptII*) transgene. The BP100 direct tandems did not show higher antimicrobial activity than BP100, and genetically modified (GM) plants constitutively expressing them were not viable. In contrast, inverted repeats of BP100, whether or not elongated with a portion of a natural antimicrobial peptide (AMP), had higher antimicrobial activity, and fertile GM rice lines constitutively expressing *bp100der *were produced. These GM lines had increased resistance to the pathogens *Dickeya chrysanthemi* and *Fusarium verticillioides*, and tolerance to oxidative stress, with agronomic performance comparable to untransformed lines.

**Conclusions:**

Constitutive expression of transgenes encoding short cationic α-helical synthetic peptides can have a strong negative impact on rice fitness. However, GM plants expressing, for example, BP100 based on inverted repeats, have adequate agronomic performance and resistant phenotypes as a result of a complex equilibrium between *bp100der* toxicity to plant cells, antimicrobial activity and transgene-derived plant stress response. It is likely that these results can be extended to other peptides with similar characteristics.

## Background

Antimicrobial peptides (AMPs) are short sequence peptides, normally less than 50 amino acid residues, reported in living systems. They are components of the defense system against pathogens in plants and animals or are produced by microorganisms in antibiosis processes (see reviews in [1-3] bacteria; [4,5] fungi; [6,7] insects; [8-10] amphibian and mammals, and [11] plants). Around 1,000 AMPs have been reported [12]. They can structurally be linear peptides (often adopting α-helical structures); cysteine-rich open-ended peptides with disulfide bridges; cyclopeptides forming a peptide ring, or pseudopeptides. AMPs offer major perspectives as a novel class of therapeutic agents, especially against fungal infections and antibiotic-resistant bacterial pathogens in humans and animals [7,9]. This great potential extends to plant disease-protection products [13-15], as substitutes of antibiotics in animal feed, biopreservatives in food, cosmetics and biomaterials, and as antifouling agents [16,17]. AMPs have proved successful as biopesticides, with commercial development of several microorganisms secreting these compounds [14].

In recent years, novel peptides have been designed, based on natural AMPs, with the aim of optimizing the activity against selected target pathogens (including microorganisms against which no AMP or antibiotic are known) while decreasing toxicity to non-target organisms and increasing stability. Short truncated compounds (minimal domain), chimerical constructions and improved sequence analogs have been reported. Examples are mellitin derivatives blocking plant viruses [18], the anti-fungal and anti-bacterial lactoferricin B derivatives [13], antifungal cecropin A and cecropin A-mellitin derived peptides [19-21], and the de novo designed antifungal hexapeptide PAF26 [22,23] and bactericide cyclic decapeptide BPC194 series [19,20].

Genetically modified (GM) plants with different degrees of resistance to pathogens have been obtained by expression of native or synthetic analogues of AMPs, either constitutively or in response to pathogen attack (reviewed in [13,14]). These include AMPs naturally produced by insects [24-29] and amphibians [30]; fungal [15,31] and plant defensins [32-36]; and modified AMP analogues such as the magainine derived Myp30 [37] and MSI-99 [38-40], MsrA3, derived from temporin A [41] and MsrA2, derived from dermaseptin B1 [42], the chimeric peptides MsrA1 and CEMA derived from cecropin A and mellitin [43,44] and the synthetic D4E1 peptide [45,46]. The expression of these AMPs in plants including tobacco, rice, potato, tomato, grapevine and cotton, have been found to give moderate resistance to relevant plant pathogenic bacteria or fungi.

Combinatorial chemistry approaches have been used to assist the design of new AMPs with superior properties. The CECMEL11 peptide library, a 125-member linear undecapeptide library contains groups of sequences with high activity against a number of reporter bacterial and fungal phytopathogenic species, several also exhibiting lowsensitivity to protease degradation and hemolytic activity [47,48]. These peptides were cecropin A-mellitin hybrids and had the structure of an amphipathic α-helix with strong positive charge at neutral pH that can facilitate electrostatic attraction to phospholipid membranes of the target microorganisms, prior to insertion of the hydrophobic face into the membrane bilayer [49,50]. In particular, BP100 (KKLFKKILKYL-NH2) displays strong, selective bactericidal activity against three plant pathogenic bacteria at micromolar concentrations, with poor antifungal properties that inhibit infections by *Xanthomonas vesicatoria* in pepper, *Erwinia amylovora* in apple and *Pseudomonas syringae* in pear [47]. The efficacy of control is comparable to standard antibiotics and it is highly biocompatible, as assessed by acute oral toxicity tests in mice [51].

The envisaged phytosanitary applications of the BP100 series of peptides makes its expression in plant systems relevant, either as potent tools to confer plant phenotypes resistance to bacterial and/or fungal pathogen species, or as biofactories of plant protection products. Phytosanitary use of these peptides against bacterial diseases of plants is limited by the high cost of production. Large-scale chemical synthesis of peptides above around 6 amino acids is only economically viable for applications of very high added value; in view of its low toxicity against animal models, the use of GM plants as AMP molecular farms could putatively be an economic alternative. Production of a number of proteins using plants as biofactories has been reported in pharmaceutical applications [52-54], and a number of companies are currently using different approaches with various proteins [55].

However, expression of AMPs in plants requires specific strategies, due to their particular properties, and has proven challenging. The 17 and 19 amino-acid long D4E1 and MsrA3 are among the shortest peptides expressed in plants [41,45]. However, a number of AMPs with relevant properties are shorter. In particular, combinatorial chemistry approaches used to engineer improved synthetic peptides are usually based on smaller lengths [19,20,22,23,47,48]. For expression in plant systems, the length of these peptides should be increased above a minimum threshold while maintaining biological properties [56]. AMPs with high antimicrobial activity have been associated with high toxicity to transgenic plant cells, and peptides with moderate activity have often been expressed, including those especially modified to decrease the antimicrobial activity (e.g. MsrA1, [44]). Foreign AMPs expressed in plants can be prone to cellular degradation by endogenous peptidases, thereby limiting the level of accumulation (see e.g. [57]). Targeting the endoplasmic reticulum (ER) has been suggested as a way to improve accumulation levels, due to either the low proteolytic activity in the lumen or the higher folding ability of ER resident chaperones [58,59]. Confining AMPs to the ER compartment would be expected to decrease their potential toxicity to the plant cell. Moreover, GM rice plants expressing ER targeted cecropin A had resistant phenotypes [29]. Interestingly, any change in the peptide sequence, such as enlargement, the introduction of hinge sequences, ER retention signal, or specific tagging, may affect the biological properties, and this would require a considerable level of screening.

To look at BP100 as a proof-of-concept of the possibility of expressing active AMPs of the BP100 series in plants, a number of BP100 derivatives were rationally designed to be produced in plants. The possible influence of these sequence modifications on the expected antibacterial activity was experimentally tested using chemically synthesized BP100 derivatives and specific bacterial growth inhibition assays. The possibility of producing BP100 derivatives with high antimicrobial activity in a plant system was investigated by stable transformation of rice, following a strategy based on constitutive expression and ER accumulation. The impact of this type of peptide on the fitness of the host plant was specifically evaluated. We envisage our results will be applicable to other short α-helical cationic peptides with high antimicrobial activity.

## Results

### Rationale of the approach - Design of BP100 derivatives suitable for expression in GM plants

BP100 is a synthetic linear polypeptide with only L-amino acids, which is compatible with standard protein synthesis in plant cells. However, BP100 is amidated at the C-terminal position [47]. An unmodified form of BP100 was chemically synthesized, retaining the same amino acid sequence. On analysis, it was found to have similar or slightly lower antibacterial activity and hemolytic activity as BP100 (Table 1), so was selected as a model undecapeptide from the CECMEL11 peptide library for AMP production in plants.

**Table 1:** Antibacterial, phytotoxicity and hemolytic activity of BP100 derivatives

**BP100 derived peptide**	**# Amino acids**	**Antimicrobial activity**					**Phytotoxicity (germination)**	**Phytotoxicity at 150** μ**M(inoculation)**	**Hemolytic activity at 150 μM**
		***Ea***	***Pss***	***Xav***	***Dc***	***Fv***			
BP100	11	2.5 - 5.0	2.5 - 5.0	5.0 - 10	5-10	<2.5	64	0.90 ± 0.05^a^	22.0 ± 2.8
unmodified BP100	11	5.0 - 10	5.0 -10	10 - 20	10 - 20	<2.5	64	0.80 ± 0.02^a^	1
BP100.1	15	2.5 - 5	2.5 - 5	1.25 - 2.5	10 - 20	<2.5	64	1.17 ± 0.09^ab^	<1
BP100.2	30	5.0 - 10	5.0 -10	5.0 - 10	>40	<2.5	32	1.15 ± 0.07^ab^	3
BP100.2i	30	2.5 - 5	2.5 - 5	1.25 - 2.5	20 - 40	2.5 - 5	16	1.32 ± 0.06^b^	4
BP100.3	45	>20	>20	10 - 20	>40	5-10	16	1.38 ± 0.04^b^	4
BP100.2mi	48	2.5 - 5	2.5 - 5	<0.6	20 - 40	2.5 - 5	16	1.46 ± 0.12^b^	5

Antibacterial activity was determined against *Erwinia amylovora* (*Ea*), *Pseudomonas syringae* pv. *syryngae* (*Pss*) and *Xanthomonas axonopodis* pv.*vesicatoria *(*Xv*) as reporter species, and against the rice pathogens *Dickeya chrysanthemi* (*Dc*) and *Fusarium verticillioides* (*Fv*).

Minimal inhibitory concentrations (MIC) are given in μM and were calculated with 10^8^ bacterial CFU/ml or 10^4^ conidia/ml. Phytotoxicity values in rice germination assays are given as minimal peptide concentrations (μM) causing significant alteration of shoot length. Phytotoxicity values in tobacco leaf inoculation assays are given as the mean of the lesion diameter (cm) and standard error upon inoculation of 150 μM peptide solutions. Letters indicate different groups in one-way ANOVA analysis and Tukey's b posttest with α = 0.05. Hemolytic activity is given as the ratio between each peptide and the reference peptide BP100 (calculated at 150 μM and given in % with confidence interval for α = 0.05 [47]).

**Figure 1:**
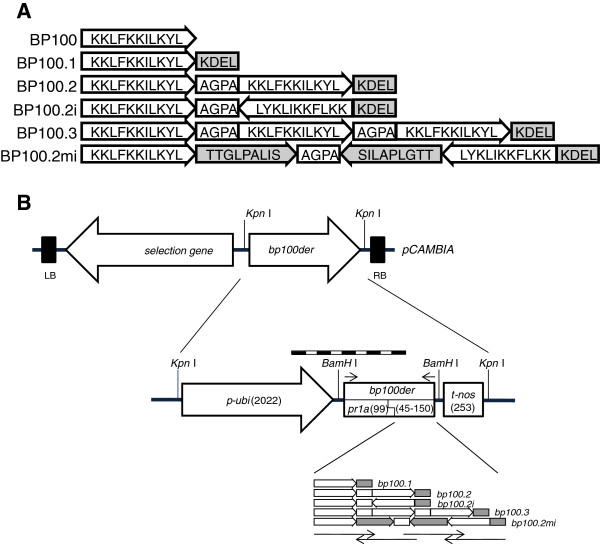
**Schematic representation of the BP100 derivatives designed and the components of transgene cassettes in plasmids used for rice transformation.(A)** White arrow, BP100; grey arrow, mellitin extension (nucleotides 19–18); white rectangle, linker sequence; grey rectangle, ER retention sequence. Arrows indicate the orientation of the sequences. **(B)Top**, schematic diagram showing the selection (*hptII*) and *bp100der* genes in a pCAMBIA1300 plant expression vector. LB and RB, *A. tumefaciens* left border and right border sequences. Expression of the *bp100der* genes is driven by the maize ubiquitin promoter (*p-ubi*), including the first exon and first intron (overall 1061 bp) and the nos terminator (*t-nos*). The hygromycin phosphotransferase *hptII* gene was used in combination with the cauliflower mosaic virus 35 S promoter and terminator sequences, *p-35 S* and *t-35 S* (overall, 2015 bp). *bp100der* length is between 2419 and 2569 bp. **Center**, regulatory and coding elements in the *bp100der* gene. The different sequence elements are not drawn to scale. Lengths (in base pairs) are indicated in brackets. Dashed box corresponds to the probe used in Southern analysis, and arrows above *bp100der* indicate RT-qPCR primers. **Bottom**, sequences encoding the BP100 derivatives: white, AGPA; grey, KDEL; light faded rectangle, BP100; dark faded rectangle, mellitin fragment. Color fading indicates the orientation of each amino acid sequence. Arrows below represent oligonucleotides (corresponding to *bp100.3*) used for recursive PCR. Restriction sites relevant for cloning are indicated.

The length of the BP100 undecapeptide, a short molecule, needs to be increased above a minimum threshold to achieve expression in plant systems. Accumulation of this bactericidal peptide in plants could potentially result in BP100 degradation by plant proteolytic activities (e.g. in intercellular spaces) and/or produce plant cell damage even though cytotoxicity and protease susceptibility were minimized during the BP100 selection procedure. This effect is typically diminished in cell compartments such as the ER, where optimal yields of recombinant proteins have often been described. Five BP100 derivative peptides, including up to three copies of BP100 in the same or in opposite orientations, were specifically designed for expression in plants and accumulation in the ER. The derivatives were 15 to 48 amino acids long (see scheme in Figure 1).

It has been previously reported [47] that single amino acid changes in the BP100 sequence have a major effect on its biological characteristics. We tested the antibacterial activity of the newly designed BP100 derivatives by *in vitro* growth inhibition of the three model plant pathogen bacteria *Erwinia amylovora* (*Ea*), *Pseudomonas syringae* pv. *syringae* (*Pss*) and *Xanthomonas axonopodis* pv. *vesicatoria* (*Xav*). The different modifications incorporated in BP100 derivatives designed for expression in plants affected the properties of the resulting peptides (Table 1). BP100.1, BP100.2i and BP100.2mi had similar or higher antibacterial activity compared to BP100, and the increase in activity was higher against *Xav* than against *Ea* and *Pss*. BP100.2mi was the most active peptide and BP100.2 and BP100.3 were the least active BP100 derivatives in this assay.

### Toxicity of BP100 derivatives

**Figure 2:**
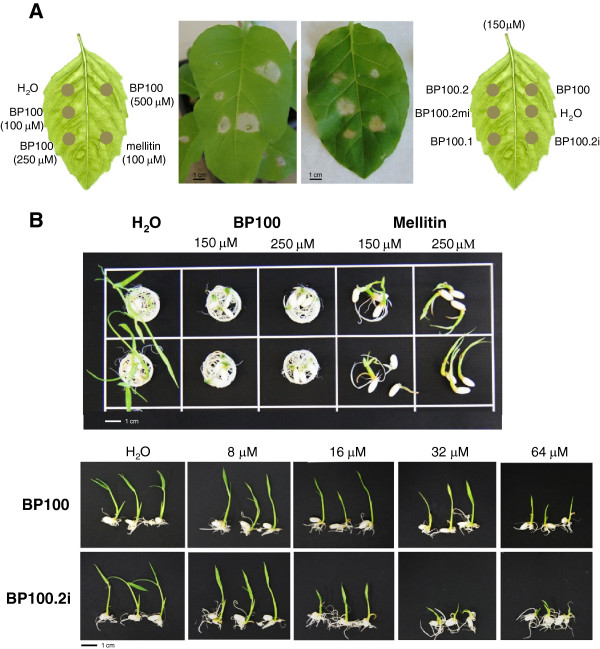
**Phytotoxicity of chemically synthesized BP100 derivatives.(A)** Representative examples of tobacco leaves, three days after inoculation with H_2_O, BP100, BP100 derivatives and mellitin. **(B)** Inhibition of germination assay. **Top**, representative examples of rice Senia seeds germinated for 7 days in H_2_O or high concentrations of BP100 and mellitin. **Bottom**, representative examples of rice Senia seeds germinated for 7 days in H_2_O or decreasing concentrations of BP100 and BP100.2i. Scale bars: 1 cm.

We initially assessed the possible toxicity to plant producer cells of BP100 derivatives by inoculation of chemically synthesized peptides in tobacco leaves in an *in planta* assay. Inoculation of 50 to 250 μM BP100.1, BP100.2, BP100.2i, BP100.3 and BP100.2mi induced lesions in tobacco leaves in a dose dependent manner (Figure 2A), with water as the negative control. The highly cytotoxic peptide mellitin was used as the positive control. BP100 derivatives produced similar or moderately larger lesions than BP100 (Table 1), while mellitin clearly produced more severe lesions at the same doses.

Further assays were carried out with rice. In a germination test, high concentrations of both BP100 and mellitin severely affected seedling development (Figure 2B). Remarkably, the effects of the two peptides were divergent. Mellitin predominantly inhibited root growth while allowing moderate shoot development. In contrast, BP100 barely effected root growth but almost completely inhibited shoot development. The phenotypic effects of BP100 derivatives were similar to those of BP100. Phytotoxic effects were quantified through shoot lengths of seedlings grown in the presence of decreasing concentrations of peptides, and minimal peptide doses causing significantly shorter seedlings were determined (Table 1). Toxic effects were dose dependent. BP100, unmodified BP100 and BP100.1 produced phenotypic effects at 64 μM and higher concentrations (one-way ANOVA *P* values at 64 μM were 0.001, 0.001 and 0.000, respectively). BP100.2 produced shorter seedlings at 32 μM and higher concentrations (one-way ANOVA *P* value at 32 μM, 0.000); and BP100.2i, BP100.3 and BP100.2mi were toxic at 16 μM and higher concentrations (for each peptide at 16 μM, one-way ANOVA *P* = 0.000) (Figure 2C).

These results demonstrated that BP100 and its derivatives were toxic to plant cells when applied at high doses. The toxicity to mammalian cells of BP100 derivatives was tentatively assessed as the ability to lyse erythrocytes in comparison to a potent hemolytic agent, mellitin. As shown in Table 1, BP100 derivatives generally had higher hemolytic activities than BP100 (except for BP100.1), and this activity seemed to increase with peptide length.

### Effect of *bp100der* transgenes on the efficiency of transformation

We assessed the feasibility of synthetic AMP production in plants using rice as the model, and on the basis of constitutive transgene expression and AMP accumulation in the ER to protect foreign peptides from potential degradation by proteolytic activities and plant cells from phytotoxic activity of peptides. To get a broad picture of the potential and possible limitations of this approach, all five BP100 derivatives were used for transformation, even though they had different characteristics (see above). Chimeric genes encoding all five BP100 derivatives (i.e. *bp100.1*, *bp100.2*, *bp100.2i*, *bp100.3* and *bp100.2mi*, collectively named *bp100der*) were synthesized and used in a series of transformation experiments designed to quantify the efficiency of the process in terms of amounts of transgenic calluses and plants that could be obtained for each BP100 derivative. A total of 1,200 rice calluses were transformed with each AMP plasmid and the empty vector, with the *hptII* selection gene and no *bp100der* sequence, and the numbers of transgenic calluses were recorded. Regeneration and development of transgenic plantlets were also monitored (Table 2).

**Table 2:** Progress of the rice transformation with transgenes encoding BP100 derivatives

**BP100 derived peptide**	Hygromycin resistant calluses*	Regenerated plants*	Acclimated plants*	Plants producing seeds*	**Overall efficiency of the process compared to control plasmid**
control	110**	80%	100%	100%	
BP100.1	43 (39%)	21 (61%)	10 (48%)	9 (90%)	10%
BP100.2	23 (21%)	0 (0%)			<1%
BP100.2i	82 (75%)	20 (30%)	5 (25%)	2 (40%)	2%
BP100.3	20 (18%)	0 (0%)			<1%
BP100.2mi	83 (75%)	8 (12%)	8 (100%)	3 (38%)	3%

Absolute numbers and percentages relative to transformation with the control plasmid are given. Control transformation data is given in absolute numbers of hygromycin resistant calluses and percentages of transgenic plants, always relative to the previous step.

*A total of 1200 calluses were transformed with each plasmid in three independent experiments, each including transformation with the control vector. Only hygromycin resistant calluses and plants from different calluses (i.e. independent transformation events) were recorded.

**Only 15 of the 110 control hygromycin resistant calluses were further processed.

Transformation with all five *bp100der* plasmids resulted in growth of hygromycin-resistant transgenic callus. However, we found major differences in the efficiency of the transformation among plasmids with sequences encoding different BP100 derivatives. Transformation with control plasmids consistently gave the highest numbers of transgenic calluses in our experimental conditions. Slightly lower numbers of calluses showing the selection phenotype were obtained with plasmids carrying *bp100.2mi* or *bp100.2i*, while much reduced numbers of transgenic calluses carrying *bp100.1* and especially *bp100.2* and *bp100.3* were identified.

To confirm the functionality of *bp100der* transgenes in callus with the selection phenotype, we assessed transgene mRNA expression in three randomly chosen transgenic calluses for each construct by RT-qPCR (RNA extraction and cDNA synthesis were systematically performed in duplicate). All analyzed calluses had been subjected to various subculture steps to minimize non-transgenic cells. In a preliminary test we assessed the mRNA levels of three reference genes; elongation factor EF1α, β-actin and 18 S RNA ribosomal genes. In all our callus samples, β-actin had the best score upon application of the GeNorm algorithm (M value = 0.48), so was selected for normalization.

All analyzed calluses expressed both selection marker and (except for those with an empty vector) the corresponding *bp100der* transgenes (Additional file 1). As expected, transgene expression levels calculated by comparison to β-actin showed great variation among the different events, with relative standard deviations (RSD) of normalized transgene mRNA copy numbers of 63% and 117% for *hptII* and *bp100der*, respectively. Compared to β-actin, the amount of selection gene mRNA in GM callus was generally 1- to 10-fold higher, and that of *bp100der* mRNA 10- to 110-fold higher. The *hptII* selection gene was similarly expressed in transgenic callus transformed with plasmids carrying different *bp100der* sequences or the empty vector (5.8 ± 3.7 -fold those of actin; one-way ANOVA *P = * 0.630). Transgenes encoding distinct BP100 derivatives were expressed at statistically similar levels in calluses exhibiting the selection phenotype (30.3 ± 35.5 -fold those of actin; one-way ANOVA *P =* 0.283), although, the lowest *bp100der* expression values consistently corresponded to *bp100.2*.

Callus with the *hptII* selection gene and *bp100.2* grew slowly, suffered necrosis and never regenerated GM plants. Although they grew normally, none of the ~20 *bp100.3-* transformed hygromycin-resistant calluses achieved plant regeneration, so no GM plants expressing this AMP (S-bp100.3) could be obtained with this approach. In contrast, plant regeneration was possible for control, *bp100.1*, *bp100.2i* and *bp100.2mi* transformants (S-hgr, S-bp100.1, S-bp100.2i and S-bp100.2mi plants, respectively), although at decreasing rates. The presence of the *bp100der* transgene was assessed by qPCR in leaf samples of all regenerated plantlets. Two plants, transformed with *bp100.1-* and *bp100.2i-*containing plasmids, were qPCR negative and were discarded. An additional plant produced a *bp100der* qPCR product of different melting temperature than expected. Sequencing of the insert proved the *bp100.2mi* sequence in this event carried a copy of *bp100.2mi* with a deletion and was also discarded. Finally, 21, 20 and 8 T0 plantlets were obtained which carried the *bp100.1*, *bp100.2i* and *bp100.2mi* transgenes, respectively (Table 2). Less than half of these T0 plantlets completed acclimatization and were fertile (although all controls did). The capacity of transgenic cells carrying *bp100der* sequences to grow as callus, regenerate plants, survive greenhouse standard growth conditions and produce viable seeds was visibly inferior to controls with the empty vector. The efficiency of the whole transformation process was only 10% the control for S-bp100.1, and as low as 2-3% for S-bp100.2i and S-bp100.2mi (no S-bp100.2 and S-bp100.3 GM plants were obtained). We concluded that *bp100der* had a negative effect on transgenic callus and plants; different *bp100der* sequences had different degrees of phytotoxicity in specific steps of the transformation process.

### Molecular and cellular characterization of transgenic plants

A total of fourteen fertile transgenic plants were obtained carrying *bp100.2i* (2 independent events), *bp100.1* (9 independent events) or *bp100.2mi* (3 independent events). As all T0 transgenic plants originated from independent calluses, they corresponded to different transformation events. For selection of representative GM events, on the basis of insert copy number and *bp100der* expression levels, genomic DNA extracted from leaf samples of S-bp100.1 and S-bp100.2mi mature T0 plants was analyzed by qPCR targeting *bp100der*, *hptII* and actin, to determine approximate transgene copy numbers. Ratios of transgene to actin copy numbers were close to 0.5 for all events, suggesting they all had single copy insertions. Additional leaf samples of the same plants were subjected to RNA extraction and RT-qPCR analysis to compare *bp100der* mRNA levels. *bp100.1* was similarly expressed in the nine different S-bp100.1 events (24.9 ± 5.4 -fold actin mRNA), and *bp100.2mi* was expressed at similar levels in all three S-bp100.2mi events (2.1 ± 0.8 -fold). Three events carrying *bp100.1* (S-bp100.1-6, S-bp100.1-9 and S-bp100.1-10) and two with *bp100.2mi* (S-bp100.2mi-1 and S-bp100.2mi-9) were selected, in addition to the two events obtained for *bp100.2i* (S-bp100.2i-5 and S-bp100.2i-42).

The seven selected GM events were self-crossed to produce the homozygous T2 generation of GM plants: three independent homozygous lines harboring the *bp100.1* transgene and two with each, *bp100.2i* and *bp100.1* were obtained. Southern blot and qPCR analyses confirmed that all had single copies of the transgene (Additional file 2).

Transgene expression was assessed by RT-qPCR in leaves of homozygous plants grown under controlled conditions and sampled at the two-leaf stage. Three biological replicates of ten plants were analyzed per transgenic event. Every transgenic line expressed the corresponding *bp100der* mRNA in their leaves. Yields of transgene mRNA were calculated by comparison to actin as the reference gene (GeNorm M value < 0.5 in these samples) (Additional file 3). As expected, the expression levels of the transgenes varied in the different events: not only copy number but also the integration site is known to influence transgene expression levels [60].

**Figure 3:**
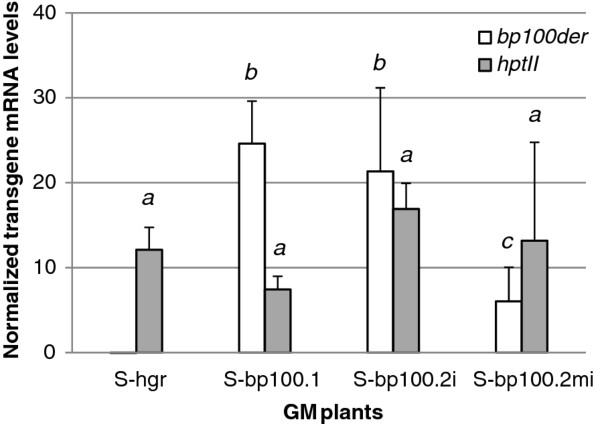
**Transgene mRNA expression levels (relative to actin) in leaves of in vitro grown homozygous T3 plants, as assessed by RT-qPCR.** Means and SD of GM events carrying every transgene are shown. Three biological replicates per GM event were analyzed, each with leaves of ten plants at the two-leaf stage. Letters indicate statistically different *bp100der* mRNA values (one-way ANOVA, Tukey's b posttest α < 0.05).

We then compared transgene expression levels between plants transformed with different *bp100der* plasmids (Figure 3). S-bp100.1 and S-bp100.2i plants expressed higher amounts of *bp100der* mRNA in their leaves than S-bp100.2mi (one-way ANOVA *P* value, 0.000). *hptII *mRNA levels were similar in transgenic plants transformed with the different plasmids, including those with the empty vector (one-way ANOVA *P* = 0.060). The presence of *bp100der* or the specific *bp100der* incorporated in GM plants did not affect the expression levels of the selection gene.

**Figure 4:**
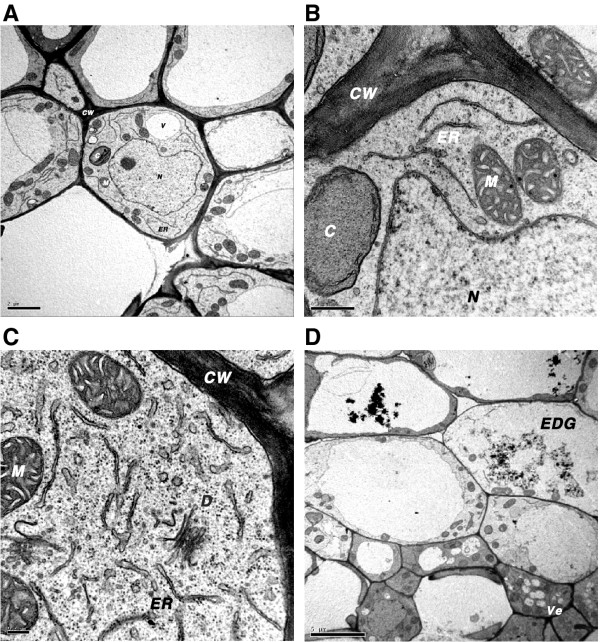
**Transmission electron micrographs of rice cells expressing *bp100der *transgenes.(A)** Control Senia vascular and surrounding, and parenchyma cells of the crown region showing normal morphology. **(B)** Detail of endoplasmic reticulum morphology of Senia cells. **(C)** Cells surrounding vascular cells, showing increased abundance of dictysome vesicles and distinct dilation of ER cisterna in S-bp100.2i cells. **(D)** Vesicles in S-bp100.2mi cells surrounding vascular cells and accumulation of electron dense granules in parenchyma cells. CW, cell wall; D, dictysome; EDG, electron-dense granules; ER, endoplasmic reticulum; M, mitochondria; N, nucleus; Ve, vesicle. Scale bars: 2 μm **(A)**, 0.5 μm **(B)**, 0.2 μm **(C)** and 5 μm **(D)**.

The *bp100der* transgenes encode highly cationic peptides and were designed with ER signal peptide and retention sequences. Accumulation of the *bp100der* product in the ER could affect the morphology of this organelle. S-bp100.2i.5, S-bp100.1.9, S-bp100.2mi.1 and Senia plants were compared at the ultrastructural level by TEM observation of the crown region of seedlings at the two-leaf developmental stage. Morphology of the ER was clearly altered in S-bp100der cells accompanying vascular cells (Figure 4), with wider and more variable intra-cisternal spaces of ER cisternae and an increase in dictysome vesicles. There was no clear morphological disorganization in organelles such as mitochondria (Figure 4C) and chloroplasts. An increasing number of vesicles were also formed, particularly in S-bp100.2mi cells, and an accumulation of numerous electron-dense granules was detected in parenchyma cells of S-bp100.2mi and S-bp100.1 (Figure 4D).

### Resistance of GM plants to pathogen infection and tolerance to oxidative stress

An indirect approach was used to obtain evidence of the production of BP100 derivative AMPs in our transgenic rice lines, testing their resistance to infection with the bacterial pathogen *Dickeya chrysanthemi* and the fungus *Fusarium verticillioides*, and tolerance to oxidative stress. Seeds from all the homozygous lines obtained were incubated in the presence of *D. chrysanthemi* and *F. verticillioides*, and germination was monitored. Homozygous plants from all lines were treated with H_2_O_2_ in detached-leaf assays.

**Figure 5:**
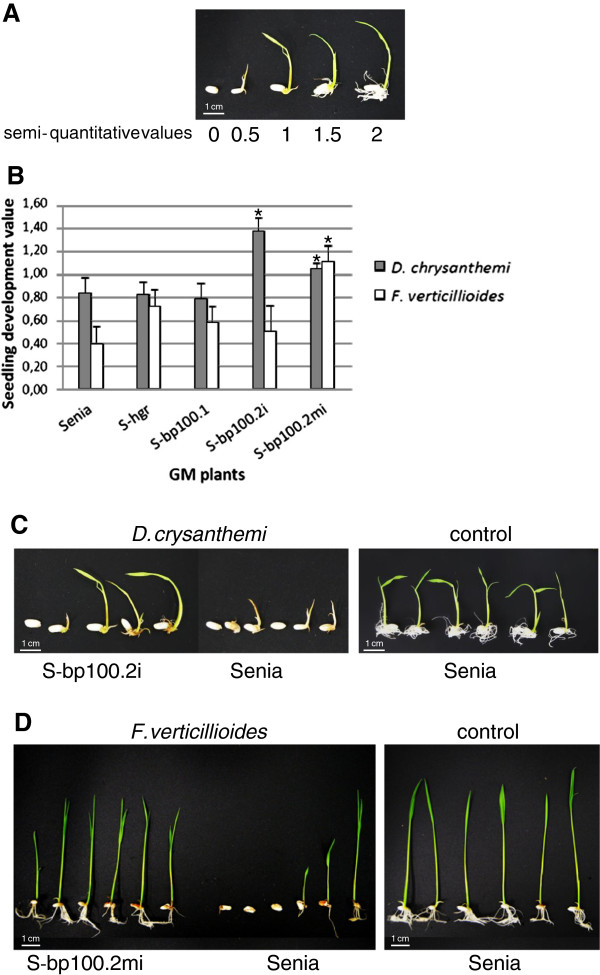
**Susceptibility to *Dickeya chrysanthemi *(*D. chrysanthemi*) and *Fusarium verticillioides *(*F*.*verticillioides *) of control untransformed Senia and S-*bp100.1*, S-*bp100.2i*, S-*bp100.2mi *and S-hgr GM plants in a germination assay.(A)** Representative examples of seedlings with different semi-quantitative development values to estimate *D.chrysanthemi* effects: 0, no germination; 0.5, cotyledon development; 1, standard shoot but no root development; 1.5, standard shoot development but short roots; and 2, standard growth. **(B)** Means and standard errors of seedling development values are represented as a function of the transgene in each line (every homozygous line obtained in this work was analyzed). Both for *D. chrysanthemi* and *F. verticillioides* susceptibility, a value of 0 indicated no germination while a value of 2 represented development similar to uninfected seeds. Asterisks indicate statistically significant differences compared to Senia (one-way ANOVA *P* values are given in the text). **(C)** Representative examples of S-bp100.2i (**left**) and Senia (**center**) seedlings grown in the presence of 10^5^ CFU *D. chrysanthemi*. Germination of Senia control seeds (**right**). **(D)** Representative examples of S-bp100.2mi (**left**) and Senia (**center**) seedlings grown in the presence of 10^5^CFU *F. verticillioides* and germination of Senia control seeds (**right**). Scale bars: 1 cm.

With Senia seeds, infection with 10^2^ to 10^6^*D. chrysanthemi *CFU resulted in progressively smaller, brownish roots and shorter shoots, with germination almost completely inhibited at the highest dose. The pathogenic effect of *D.chrysanthemi*was estimated using a semi-quantitative scale (Figure 5A), and the values recorded seven days after infection ranged from 1.9 (negative control) to 0.4 (10^6^ CFU).

We selected 10^5^ CFU (with a value of 0.8) to assess susceptibility of S-bp100der seeds to this pathogenic bacterium. S-bp100.1 and S-hgr lines had similar sensitivity values as Senia (one-way ANOVA *P* values, 0.664 and 0.953, respectively), indicating that *hptII *or *bp100.1* did not significantly modify resistance to this bacterial pathogen. In contrast, S-bp100.2i and S-bp100.2mi lines were more resistant than untransformed plants (one-way ANOVA *P* values, 0.004 and 0.036, respectively) (Figure 5B and 5C), signifying that *bp100.2i* and *bp100.2mi* transgenes decreased rice susceptibility to *D.chrysanthemi*.

Seven days after *F. verticillioides* inoculation of Senia seeds, a cottony mycelium covered the surface of the remaining seed and seedlings showed severe growth effects. The primary embryonic root was shorter than that of uninfected controls, with a brownish tone, and there was little or no crown root production. Shoots were shorter in infected seedlings. The *F. verticillioides* pathogenic effect was estimated through a semi-quantitative index that reflected shoot height and root development (number and length of crown roots, and length of primary root).

There were clear differences in the effects of this fungus on plants harboring different transgenes. Those transformed with the control plasmid, *bp100.2i* and *bp100.1* had major morphology alterations with susceptibility values similar to Senia (one-way ANOVA *P* values, 0.562, 0.697 and 0.278, respectively). In contrast, all events harboring *bp100.2mi* had long shoots and well developed root systems (Figure 5D), which resulted in higher resistance values (one-way ANOVA *P* = 0.005). This suggested that rice was protected against infection with *F. verticillioides* by *bp100.2mi *transgene but not *hptII*,*bp100.1 *or *bp100.2i*.

Automated growth curve analysis demonstrated that the presence of BP100 derivatives in the culture broth resulted in inhibition of *D. chrysanthemi* and particularly *F. verticillioides* growth in a concentration-dependent manner. The MIC values for the relevant BP100.1, BP100.3 and BP100.2mi peptides ranged from 10 to 40 and <2.5 to 5 μM, respectively (Table 1), which is indicative of their activity against this microorganism.

**Figure 6:**
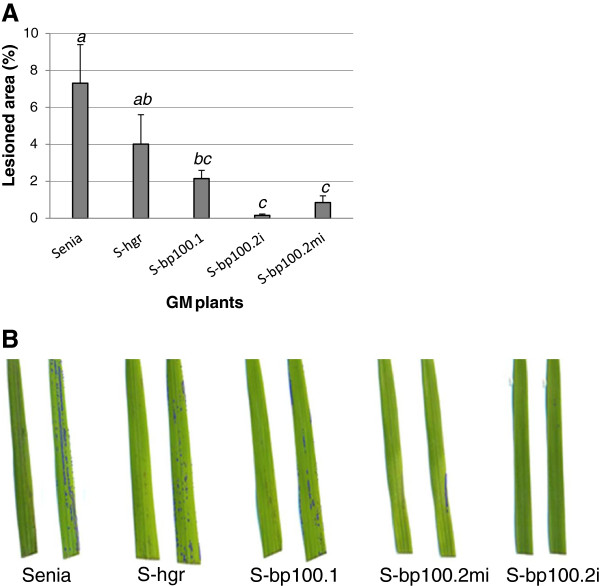
**Tolerance to H**_**2**_**O**_**2 **_**treatment (oxidative stress) of control untransformed Senia and S-*bp100.1*, S-*bp100.2i*, S-*bp100.2mi *and S-hgr GM plants in a detached leaf assay.(A)** Ten plants from every homozygous line obtained were analyzed. Means and standard errors of the percentages of affected areas are represented as a function of the transgene in each line. Letters indicate statistically significant differences (one-way ANOVA, Tukey’s-b posttest with α value < 0.05). **(B)** Representative examples of leaves subjected to H_2_O_2_ treatment. The right of each pair, labeled in blue, shows NBT stained areas used to calculate percentages of lesion areas (APS assess tool).

After treatment with H_2_O_2_, Senia untransformed leaves had the widest NBT stained leaf surface (7.3% ± 5.9), indicating accumulation of superoxide radicals (O_2_^-^). S-hgr rice lines carrying *hptII* but no *bp100der* sequences had slightly lower, statistically undistinguishable, NBT staining values (4.0% ± 3.9). NBT staining was somehow lower in S-bp100.1 lines (2.2% ± 2.0), but one-way ANOVA indicated they were similar to S-hgr (*P* = 0.408), suggesting that there was no clear improvement of stress tolerance with the *bp100.1* transgene compared to control leaves. The accumulation of O_2_^-^ was considerably lower in S-bp100.2i and S-bp100.2mi rice lines than in untransformed and S-hgr control plants (0.2% ± 0.1 and 0.3% ± 0.7 stained area, respectively; one-way ANOVA *P* = 0.001; two groups in Tukey’s b posttest α = 0.05) (Figure 6), indicating *bp100.2i* and *bp100.2mi* transgenes resulted in increased tolerance to oxidative stress.

### Performance of GM rice plants with *bp100der* transgenes

Plants from all S-bp100.2i and S-bp100.2mi homozygous lines obtained in this work were compared with control Senia in terms of agronomic parameters. For each line, three replicates of 10 plants each were analyzed. Growth and yield of control plants were as normal in our culture conditions. No significant differences were found between independent replicates of any particular GM line, and the different lines harboring the same *bp100der* transgene gave similar values for all studied parameters (one-way ANOVA *P* values above 0.05).

Slight differences were observed between control plants and those with different transgenes. S-bp100.2i plants were shorter than Senia (observations on 08/25/10), with mean height and standard error values of 90.6 ± 0.9 and 105.3 ± 1.1 cm, respectively (Additional file 4). In contrast, S-bp100.2mi plants were a similar height to the controls (107.8 ± 0.9 cm) (one-way ANOVA *P =* 0.000, two groups upon Tukey’s b posttest with α = 0.05). All lines had the same amount of tillers per plant (7.9 ± 0.2; one-way ANOVA *P =* 0.854). Leaf chlorophyll content was measured during plant growth and no significant differences were observed between S-bp100.2i and Senia lines. S-bp100.2mi exhibited slightly higher values, although they were all in a narrow range (42.8 to 44.6 spad units, observations on 08/25/10) (one-way ANOVA *P =* 0.012, two groups on Tukey’s b posttest with α = 0.05).

**Figure 7:**
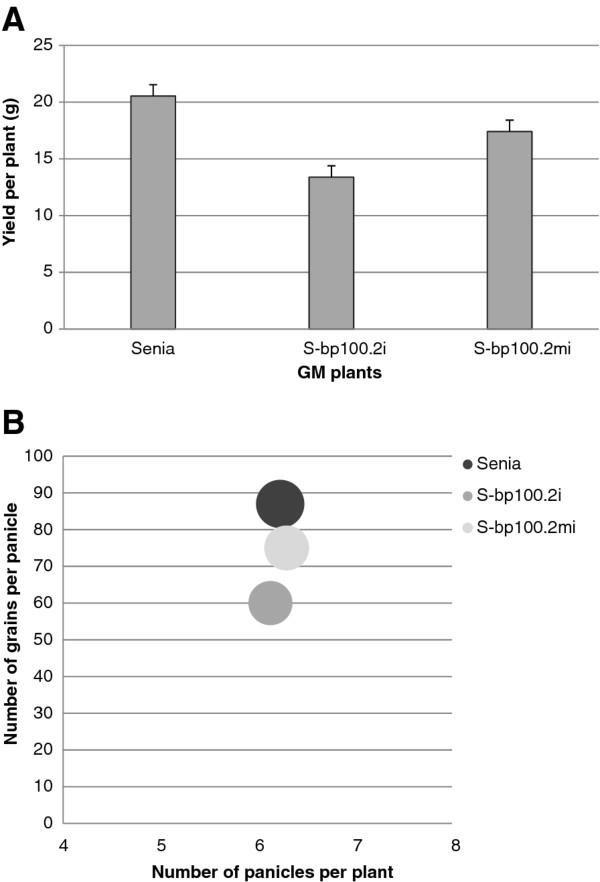
**Comparison of agronomic characteristics of untransformed Senia, S-bp100.2mi and S-bp100.2i rice plants (T3 homozygous lines).(A)** Yield of the different plants as evaluated by the weight of all panicles per plant at harvesting stage. Letters indicate statistically significant differences (one-way ANOVA, Tukey’s-b posttest with α value < 0.05). **(B)** Dispersion plot showing the number of grains per panicle (X-axis), number of panicles per plant (Y-axis) and weight of 100 grains (circle sizes are proportional to the weight of 100 grains). For S-bp100.2i and S-bp100.2mi, data from all the different events were merged.

The Senia control line had better yield, corresponding to the weight of all panicles in a plant, than lines harboring *bp100der* sequences, and S-bp100.2mi had better yield than S-bp100.2i (Figure 7A). We refined our yield data by independently considering the number of panicles per plant, number of grains per panicle and grain weight. Senia grains were heavier than those of S-bp100.2mi and S-bp100.2i lines (one-way ANOVA *P = *0.05), although overall differences were in a very narrow range (3.6 to 3.8 g/100 grains), as expected. As shown in Figure 7B Senia, S-bp100.2i and S-bp100.2mi had similar numbers of panicles per plant (mean and standard error, 6.2 ± 0.1 panicles/plant), but S-bp100.2mi and especially S-bp100.2i had less grains per panicle, which explained the lower yields.

Complementary experiments were carried out under similar conditions to compare the performance of S-hgr lines (with the *hptII* selection gene) and untransformed Senia. A total of 5 independent S-hgr events were evaluated. Four were statistically undistinguishable from Senia in terms of all analyzed parameters. One single S-hgr line had similar chlorophyll contents to Senia but it was slightly smaller (99.9 ± 0.6 cm) and had more tillers (9.9 ± 0.4 tillers per plant). There were more panicles, with fewer grains per panicle but of the same weight as in Senia, which gave rise to the same yield as untransformed lines (20.7 ± 0.7 g/plant). These were considered event dependent features.

## Discussion

Several synthetic linear undecapeptides of the CECMEL11 peptide library are of increasing interest for future development of fungicides and bactericides against plant pathogens [51]. The linear peptide BP100 is of particular interest due to its properties against major bacterial plant pathogens. Biotechnological production of this short, highly positively charged (pI = 11.02), α-helical amphipathic peptide, which could also damage producing plant cells, is challenging. The aim of this research was to investigate the feasibility of expressing transgenes encoding BP100-derived AMPs in transgenic plants using rice as the model host, particularly focusing on the putative impact on the fitness of the host plant. BP100 was used as a model for short cationic AMPs, which are recognized as a remarkable source of active substances with many different applications in diverse sectors besides plant protection [61]. We established the strategy of transgenic peptide accumulation in ER both to minimize the putative toxic effect and to protect it from plant proteases [29,57,62]. The length increase was addressed by designing larger peptides based on the BP100 sequence.

We found that any modification incorporated in BP100 derivatives designed for plant expression altered their properties in terms of antibacterial activity and range of target bacterial species. This is in agreement with previous data on how subtle changes in a peptide sequence of a given length influence antimicrobial activity [19,23,47], and with reports identifying length and sequence among the most relevant factors for biological activity of peptides [63]. It is therefore necessary to assess the biological activity and other relevant properties of any derivative of a given peptide prior to expression in plant systems.

The presence of the C-terminal KDEL element does not result in loss of antibacterial activity (see particularly the comparison between unmodified BP100 and BP100.1), which makes the strategy of peptide accumulation in ER possible. Size increase based on BP100 tandem copies did not result in a proportional improvement of activity, but tended to reduce it. In contrast, peptides with two units in inverted orientation (BP100.2i and BP100.2mi, each unit of the latter elongated with a mellitin fragment) had higher activity than BP100 against the three reporter bacteria. This increase was especially relevant against *Xav*, demonstrating that sequence variations had an effect on target specificity, which could be related to membrane composition. In view of the antimicrobial activity of these BP100 derivatives, they were considered good candidates to evaluate the possibility of expressing synthetic, highly active antimicrobial peptides in plants.

Monitoring the *Agrobacterium* based transformation process showed that *bp100der *transgene products exerted a toxic effect on constitutively producer plant cells, although BP100 has very low toxicity in hemolysis tests [47] and in a mouse model [51]. The complete transformation process of *bp100der* genes was undoubtedly inefficient, below 10% of control transformations (mean value for all 5 *bp100der* sequences, 3.2% ± 4.2). Parallel transformation with the empty vector was as expected, so excluding technical errors. Remarkably, no GM plants were obtained carrying *bp100.2* or *bp100.3*, indicating transformation efficiencies of  ≤1% that of the empty vector. AMP expression in plants has been accomplished for numerous peptides and host species ( [13,14] and references therein [24-26,32-34]). Large numbers of GM rice events accumulating *Aspergillus giganteus* antifungal protein Afp [31] or cecropine A [29] have been reported with normal phenotypes. Recently, thanathin expression has been reported to have very few harmful effects to host plant cells [26]. However, to our knowledge this is the first report on detailed comparison of transformation efficiencies of different putatively toxic transgenes, as the objective is typically to obtain a few transgenic events and the impact of transgene expression at different stages of GM plant production is not addressed.

Not only did transformation with different *bp100der* sequences have different overall efficiency values but the effectiveness of specific steps during the transformation process varied. As an example, reasonable numbers of calluses harboring *bp100.2mi* and *bp100.2i* were obtained (around 80% of the control with only *hpII*) whereas only around 20 - 40% the expected was obtained with *bp100.1* and* bp100.3*. The *bp100der* transgene mRNA levels in callus could partially explain these results, with somewhat higher expression levels (e.g. *bp100.1* and *bp100.3*) in some way compromising host cell fitness. The number of calluses transformed with *bp100.2 *was low and they suffered necrosis while expressing the transgene at the lowest *bp100der* levels. These phenomena can be interpreted on the basis of the different properties of the specific BP100 derivatives: lower levels of *bp100.2* product seem to be more toxic to the host cell than higher levels of other *bp100der* products.

The number of GM calluses regenerating plants was above 60% that of the control for *bp100.1* but extremely low for *bp100.2mi*. In contrast, all regenerated S-bp100.2mi plants but only one fourth of the S-bp100.2i plants survived the acclimatization stage, and virtually all S-bp100.1 plants in the greenhouse produced seeds but less than half S-bp100.2i and S-bp100.2mi developed and were fertile. As the *bp100der* transgene and mRNA were present in all plants obtained, regeneration of plants mainly from transgenic calluses having lost the *bp100der* transgenes could be discarded. However, for *bp100.2mi *only GM plants with low transgene expression levels were viable. Diverse *bp100der* transgene products seem to have different toxic effects on the producing cells, in particular affecting callus growth and capacity to regenerate plants, plant survival and/or development under standard greenhouse conditions. This is in agreement with the different characteristics of chemically synthesized BP100 derived peptides. Interestingly, length increment based on BP100 tandem repeats did not result in a proportional increase in activity but it greatly increased toxicity to the producer cell. This is in contrast to previous reports associating major antibacterial activity of cationic peptides to higher toxicity to eukaryotic cells [64].

Toxicity of BP100 derivatives to plant cells was confirmed in rice germination and tobacco leaf inoculation assays by comparison of the highly cytotoxic peptide, mellitin. BP100 phytotoxic effects were less intense than those of mellitin and they affected different biological pathways. BP100 reduced shoot development in seedlings while root growth was deficient with mellitin. The five BP100 derivatives had similar phytotoxic effects to BP100 (including BP100.2mi, with part of the mellitin sequence), in some cases with moderately higher intensity. Red blood cell-based assays have been used as an approach to estimate peptide toxicity to mammalian cells [65,66]. The hemolytic activity of the BP100 derived peptides was parallel to phytotoxic activity, with a tendency to increase with peptide length even though mellitin is just 26 amino acids long. Remarkably, toxicity to erythrocytes and plant cells could only be detected upon application of high peptide doses (i.e. 160 to 10-fold target pathogen MIC values). The mechanism of action of AMPs such as BP100 is based on electrostatic attraction and subsequent interaction with cell membranes [50,67]. This property has been exploited by using the BP100 sequence to internalize fused reporter proteins into eukaryotic cells [68]. Differences in membrane lipid composition between bacterial and eukaryotic cells (absence of acidic phospholipids and presence of sterols) drastically reduce susceptibility of eukaryotic cells to many cationic peptides [69] and explains the specific antimicrobial properties of BP100 derivatives. Biologically synthesized *bp100der* products are expected to accumulate at lower concentrations than those exerting visible phytotoxicity. Phytotoxicity of BP100 derivatives could explain the reduced transformation efficiencies of *bp100der* transgenes. The *bp100der* products may cause stress in S-bp100der lines, but its expression certainly can be compatible with moderately decreased fitness and increased pathogen resistance phenotypes (see below).

The possibility of obtaining transgenic rice constitutively expressing *bp100der* transgenes cannot be directly inferred from the capacity to lyse erythrocytes, damage tobacco leaves upon inoculation or affect seedling development. Transgenic plants were obtained for expression of BP100 derivatives displaying high toxicity values in our assays (BP100.2mi and BP100.2i) but not e.g. BP100.2, with lower values. Exogenous application of peptides at very high concentrations causes these toxic effects, whereas the vulnerability of host plant cells to biologically synthesized *bp100der* products may differ, as these are produced within the plant cell and are likely to accumulate in a specific cell compartment.

Fertile GM lines with a correctly incorporated and constitutively expressed single copy of the transgene were obtained for some *bp100der* sequences. The specific chemical properties of BP100 (e.g. high isoelectric point, poor antigenicity, poorly detectable by mass spectrometry) make it extremely difficult to detect and purify from plant cellular material. We are currently working on this, but no operative protocols to directly detect BP100 derived peptides in plant tissues are yet available. In addition, among the prolific number of reports in the current literature on GM plants expressing AMPs only a few gave direct confirmation of AMP production [25,40,41,43]. Nevertheless *bp100der* mRNA expression in GM plants, low transformation efficiencies and other phenotypic evidences (ultrastructural, oxidative stress and pathogen resistant phenotypes) indirectly demonstrate the production of BP100 derivative molecules in these transgenic plants.

S-bp100der cells exhibit altered ER morphology, with distinct dilation of cisterna and abundant dictysome vesicles. Taking into account that *bp100der* sequences include signal peptide and ER retention motifs, these observations agree with synthesis of *bp100der* products and accumulation in this organelle. Additionally, numerous vesicles and electron dense granules were observed in S-bp100.1 and especially S-bp100.2mi cells. Disruption of the ER structure and the presence of increased vesicles or small vacuoles and in some cases, electron dense granules have been associated to exposure of plant cells to excess (toxic) levels of heavy metals [70,71]. This means that these observed morphological characteristics could be related to the cationic nature (as heavy metals) and/or toxic character of BP100 derivatives. Toxicity caused by the expression of *bp100.2mi* seems to be higher than that of *bp100.2i*.

Our indicator bacterial species *Ea*, *Xav* and *Pss* are not pathogens of rice so cannot be used to assess resistance of our GM rice plants. However, several antimicrobial proteins in GM plants have been reported to confer protection against a wide range of abiotic and biotic stress conditions. Transgenic expression of the *C. annuum* antimicrobial protein CaAMP1 in pepper has been shown to confer broad-spectrum resistance against pathogens [72]. In particular, overexpression of cecropin A in rice has been shown to be effective against the fungal blast *M. grisea*[29] and other conditions such as oxidative stress [73]. Expression of chimerical peptides including cecropin A has been found to give GM potatoes broad-spectrum antimicrobial activity [44], and other cationic α-helical peptides, such as the MsrA2 derivative of Dermaseptin B1 from *Phyllomedusa bicolor*, have been reported to confer resistance to a variety of fungal and bacterial phytopathogens in transgenic potato plants [42].

Activity of chemically synthesized BP100, BP100.1, BP100.2i and BP100.2mi peptides against the soft rot pathogen *D. chrysanthemi* (syn. *Erwinia chrysanthemi*[74]) was dose-dependent. Although they are mainly antibacterial, BP100 derivatives additionally exhibited antifungal activity against *F. verticillioides*, associated with the bakanae disease of rice [75]. This correlated well with the increased resistance of S-bp100.2mi lines to *F. verticillioides* and S-bp100.2mi and S-bp100.2i lines to *D. chrysanthemi*. Biotic and abiotic stresses are typically associated with the rapid production of reactive oxygen species (ROS), including H_2_O_2_ and O_2_^-^. ROS are known to play dual roles; they play a central role in the regulation of biological processes such as stress response, hormone signaling and development, but high ROS levels have been implicated in the damaging effects of various environmental stresses [76]. S-bp100.2mi and S-bp100.2i vegetative tissues subjected to oxidative stress showed decreased accumulation of O_2_^-^ radicals, suggesting an increased ability to scavenge ROS. Overexpression of *bp100.2mi* and *bp100.2i* led to enhanced resistance to bacterial and fungal pathogens; and improved tolerance to oxidative stress.

The resistant phenotype of S-bp100.2mi and S-bp100.2i was not solely an outcome of the selection gene and/or the transformation itself as shown by (i) the different susceptibility phenotypes exhibited by S-bp100.2i and S-bp100.2mi lines; and (ii) the lack of resistance of untransformed Senia and the GM S-hgr and, unexpectedly, S-bp100.1 lines. Chemically synthesized BP100.1 was active against our indicator bacterial species, *D. chrysanthemi* and *F. verticillioides*, but *bp100.1* was the less phytotoxic *bp100der* transgene. Taking into account its extremely small length (15 amino acids), we can speculate that this BP100 derivative was rapidly degraded in S-bp100.1 plants and/or produced in a modified form (e.g. not properly processed).

The expression of foreign genes in plants can trigger the activation of plant defense mechanisms normally activated only during pathogenesis [77]. Several resistant GM rice lines such as those constitutively expressing cecropin A [73], the antifungal protein AFP [78] or a pathogenesis-related (PR) protein [79] have been reported to overexpress endogenous defense genes in the absence of the pathogen. This means we cannot rule out transgene dependent overexpression of stress genes being the cause of the resistance phenotypes observed in S-bp100.2i and S-bp100.2mi lines. However, reverse transcription coupled to real-time PCR analyses of PR1b and PR5 coding genes [GenBank: U89895, X68197, widely used indicators of induction of plant defense responses [80]] showed they were expressed at similar levels in leaves of S-bp100.2i and S-bp100.2mi GM when compared to Senia *in vitro* grown plants. Additionally, plants overexpressing transgenes encoding ER driven proteins have been reported to constitutively express the unfolded protein response (UPR), activated by misfolded protein accumulation in the ER [73]. The UPR has recently been associated to plant resistance to abiotic stress and pathogen attacks ( [81], and references therein), so the accumulation of transgene products (expected to be highly cationic peptides) in ER could result in increased-resistance phenotypes. In a preliminary experiment we showed that S-bp100.2mi and S-bp100.2i were somewhat resistant to the causative agent of rice blast *M. grisea*, while we could not detect clear *in vitro* activity of the corresponding peptides against *M. grisea* spores (data not shown). S-bp100.2i and S-bp100.2mi pathogen resistant and oxidative stress-tolerant phenotypes support the expression of active antimicrobial peptides in these plants, irrespective of whether these phenotypes are peptide-direct effects or indirectly derived from transgene dependent activation of other cellular pathways. The latter seems to have a role in S-bp100.2i decreased susceptibility to *F. verticillioides*, taking into account that BP100.2mi and BP100.2i have similar MIC values against this pathogen, whereas evidence from ultrastructural analysis and mRNA expression suggest the *bp100.2mi* product exerts higher toxicity on host cells than the *bp100.2i* product.

Although with low efficiency, we showed the feasibility of producing rice lines expressing at least some *bp100der* sequences (e.g. S-bp100.2i and S-bp100.2mi) and indirectly demonstrated the production of BP100 derivative molecules. Thorough assessment of agronomic characteristics showed the nutritional status of these GM plants was unaffected by *bp100der* transgenes, as demonstrated by leaf chlorophyll content: chlorophyll content has been directly related to nitrogen content, which is considered an indicator of plant nutritional status. Although S-bp100.2i plants were around 10% shorter than expected, the greatest reduction in the measured parameters was the number of grains per panicle in GM (especially S-bp100.2i) compared to control lines. It is well known that stress conditions have an effect on the numbers of grains per panicle, so it could be speculated that this was a consequence of the phytotoxic effect of BP100 derivative accumulation in plant cells. The slight reduction in grain weight is in agreement with it being a well conserved character under different cultural conditions. Despite this, the overall performance of rice lines expressing certain *bp100der* transgenes strongly resembled comparable untransformed lines. The best case was S-bp100.2mi, with similar vegetative features and only around 10% grain yield losses compared to Senia.

## Conclusions

We found that transformation of rice with genes encoding α-helical cationic AMPs such as those derived from the synthetic 11 amino acids-long BP100 had, in many cases, a highly negative effect on the efficiency of transformation. Remarkably, GM lines constitutively expressing certain *bp100der* sequences were produced with resistance phenotypes and minimal impact on agronomic performance. BP100 tandem copies could not be constitutively expressed in GM plants, but sequences encoding BP100 inverted repeats either elongated or not with portions of a natural AMP (*bp100.2mi* and *bp100.2i*) greatly diminished transgene undesired effects. It is likely that GM plants constitutively expressing *bp100der* transgenes and displaying resistance to representative plant pathogens and fit phenotypes are the result of a complex equilibrium between *bp100der* product phytotoxicity, antimicrobial activity and transgene-dependent plant stress response. This approach could be considered feasible for expressing synthetic AMPs in plants either to establish disease resistant plants or to facilitate molecular farming.

## Methods

### Chemical synthesis of BP100 derived antimicrobial peptides

BP100 derivative peptides were designed with increasing lengths and inclusion of an ER retention signal (KDEL four-amino acid sequence). They included two or three copies of BP100, with a four amino-acid linker element, AGPA, which structurally links the α-helical active domain and the C-terminal domain in natural cecropin A [82]. One of the designed peptides was further elongated by adding a portion of mellitin (amino acids 10 to 18), another another natural AMP. Two BP100 derivatives were designed with monomeric units in opposite orientations (see scheme in Figure 1).

BP100 derived peptides were manually synthesized by the solid-phase method using Fmoc-type chemistry as previously described [47]. Briefly, Fmoc-Rink-MBHA resin (0.64 mmol/g) was used as solid support, and side-chain protection was performed with *tert*-butyloxyrocarbonyl for Lys and Trp, and *tert*-butyl for Tyr. HBTU and DIEA (3 equiv. each) mediated couplings of Fmoc-amino acids (3 equiv.) in *N,N-dimethylformamide* (DMF), monitored by the ninhydrin test. After removal of the Fmoc group with piperidine-DMF (3:7) the peptidyl resin was washed with DMF. On completion of the sequence, peptides were cleaved from the resin with TFA-H_2_O-TIS (95:2.5:2.5) and dissolved in H_2_O and lyophilized after TFA evaporation and diethyl ether extraction. HPLC was used to assess their purity, which was above 90% in all cases. Peptide identity was finally confirmed by electrospray ionization mass spectrometry.

## In vitro assessment of the properties of BP100 derivatives

### Antimicrobial activity

Peptides were solubilized in sterile Milli-Q H_2_O to a concentration of 1 mM and filter sterilized through a 0.22 μm pore filter. The plant pathogenic bacterial strains *Erwinia amylovora* PMV6076 (*Ea*, Institut National de la Recherche Agronomique, Angers, France), *Pseudomonas syringae*pv. *syringae* EPS94 (*Pss*, Institut de Tecnologia Agroalimentària, Universitat de Girona, Spain), *Xanthomonasaxonopodis* pv. *vesicatoria* 2133–2 and *Dickeya chrysanthemi* 1552-10-1 (*Xav*, *Dc*, Instituto Valenciano de Investigaciones Agrarias, Valencia, Spain) and the plant pathogenic fungal strain *Fusarium verticillioides* A-999 (*Fv*, ex-type *Fusarium* spp. collection deposited at the Department of Plant Pathology, Kansas State University Manhattan, Kansas, USA, provided by R. Jiménez-Diaz) were used for antimicrobial activity tests. Bacterial strains were stored at −80°C in Luria Bertani (LB) broth with glycerol (20%). After 24 h (*Ea*, *Pss* and *Dc*) or 48 h (*Xav*) growth in LB agar at 25°C, bacterial colonies were scraped off the surface, suspended in sterile H_2_O and adjusted to 10^8^ CFU/ml. The fungal strain was stored at 4°C in the particulate solid carrier perlite, on fresh potato dextrose agar (PDA). *Fv* cultures were prepared in potato dextrose broth (PDB) and incubated for one week at 25°C in the dark in a rotary shaker at 125 rpm. They were filtered through several layers of sterile cheesecloth to eliminate macroconidia and mycelial growth. The effluent was centrifuged at 8,000 rpm for 20 min at 4°C and the pellet was suspended in sterile water. Microconidia concentration was determined in a counting chamber.

Serial dilutions of synthetic peptides were prepared at 400, 200, 100, 50, 25, 12.5 and 6 μM. Twenty μl aliquots were mixed in a microtiter plate with 20 μl bacterial suspensions (10^8^ CFU/ml) and 160 μl trypticase soy broth (TSB, BioMèrieux, France), or 80 μl fungal suspensions (10^4^ conidia/ml) and 100 μl of double-concentrated PDB containing 0.006% (w/v) chloramphenicol. Three replicates were carried out per microbial strain, peptide and concentration. In the experiments, water was used as the positive control instead of the peptide, or as the negative control instead of the microbial suspension. Microplates were incubated at 25°C for 48 h (bacterial strains) or 22°C for 6 days (*Fv*), and optical densities at 600 nm were recorded hourly (every two hours for *Fv*) after 20 s shaking. Two experimental replicates were performed. The lowest peptide concentration showing negative microbial growth at the end of the experiment was taken as the minimal inhibitory concentration (MIC).

### Phytotoxic activity

For each treatment, a total of 12 surface-sterilized Senia seeds were germinated in 500 μl water or peptide solution at the appropriate concentration (0, 8, 16, 32 and 64 μM) in a culture chamber (28 ± 1°C with a photoperiod of 16 h light / 8 h dark under fluorescent Sylvania Cool White lamps) for seven days. Seedling morphology was recorded and shoot length was measured. Mellitin and BP100 were also tested at 125 and 250 μM.

Tobacco (*Nicotiana benthamiana*) plants were grown from seed in a heated glasshouse and used between 20 and 30 days old. One hundredμl chemically synthesized peptides at 50, 100, 150 or 250 μM were inoculated into the mesophylls of fully expanded tobacco leaves (previously wounded with a needle), using a syringe without needle, and plants were kept at standard glasshouse conditions for three days. Up to six independent inoculations were carried out in a single leaf, and at least three independent inoculations were performed per peptide and concentration, randomly distributed in different leaves and plants. Toxicity was measured as the lesion diameter.

### Hemolytic activity

Hemoglobin release from erythrocyte suspensions of fresh human blood (5% v/v) was used to evaluate hemolytic activity of synthetic peptides [47]. Briefly, aseptically collected blood (BD vacutainer K2E System with EDTA, Belliver Industrial State, Plymouth, UK) was centrifuged at 6,000 g for 5 min, washed with 10 mM TRIS, 150 mM NaCl, pH 7.2 and suspended in the same buffer. Fifty μl of peptide solutions at 300 μM were mixed with 50 μl aliquots of erythrocyte suspensions and incubated for 1h at 37ÂºC with shaking. After centrifugation, the supernatants were transferred to microplate wells with 80 μl H_2_O and the 540 nm absorbance was monitored with a Bioscreen plate reader. Hemolysis percentages were calculated relative to mellitin and TRIS buffer.

### Construction of the chimeric *bp100der* genes and plant expression vectors

A wide variety of sequence-dependent factors influence gene expression levels and we took several critical ones into account. The deduced DNA sequences encoding BP100 derivatives were codon-optimized for more preferred codon usage in rice plants [83]. The distribution of codon usage frequency along the sequence was good, with codon adaptation index (CAI) values from 0.82 to 0.87 (CAI > 0.8 indicates a high level of expression). All five sequences had average GC contents close to 50% and no peaks outside the 30-70% range were found in 60 bp windows, which could reduce mRNA half-life. No internal ribosomal binding sites or other putatively destabilizing cis-acting elements were found. The DNA sequences encoding BP100 derivatives are shown in Additional file 5.

The signal sequence from the tobacco pathogenesis related protein PR1a [84] was fused to each AMP coding sequence to drive the BP100 derivative peptides to the secretory pathway. With the KDEL C-terminal sequence, it has been successfully used to drive foreign proteins to the ER in rice var. Senia [29]. Chimerical genes encoding BP100.1, BP100.2 and BP100.3 (*bp100.1*, *bp100.2* and *bp100.3* genes) were fully synthesized by recursive PCR [85] in a single reaction. Four oligonucleotides (i.e. two in sense and two in antisense orientations, Table 3 and Figure 1) were designed to cover the whole *bp100.3* sequence with partial overlapping. The outermost oligonucleotides incorporated *BamHI* restriction sites for subsequent cloning. They were synthesized on an Applied Biosystems 394 DNA synthesizer according to [31]. Recursive PCR was carried out in a final volume of 100 μl with 30 pmol external and 0.2 pmol internal primers, 300 μM dNTPs, 1x reaction buffer, 1.5 mM MgCl_2_ and 1.75 units Expand High Fidelity DNA polymerase (Roche Diagnostics Corporation, Indianapolis USA). Reaction conditions were as follows: initial denaturing step (3 min 94°C); 10 cycles of 15 s at 94°C, 30 s at 45°C and 3.5 min 72°C; 20 cycles of 15 s at 94°C, 30 s at 58°C and 3.5 min 72°C; and a final elongation step of 10 min at 72°C. Due to the repetitive nature of *bp100.3*, this amplification consistently resulted in a mixture of three bands with lengths compatible with sequences containing one, two and three tandemly repeated *bp100* units. Recursive PCR products were cloned using the pGEM®T-Easy system (Promega, Wisconsin, USA) according to the manufacturer’s instructions. Individual clones were subjected to PCR with primers *PR1a_for* and *KDELbam_rev* (Table 3) to individually amplify *bp100.1*, *bp100.2* and *bp100.3*. The PCR was in a final volume of 50 μl 1x reaction buffer with 1.5 mM MgCl_2_, 200 nM primers, 200 μM dNTPs and 1 unit Expand High Fidelity DNA polymerase (Roche Diagnostics Corporation). Reaction conditions were 3 min at 95°C; 40 cycles of 30 s at 95°C, 30 s at 58°C and 30 s at 72°C; and 10 min at 72°C. Synthetic *bp100.2i *and *bp100.2mi* chimerical genes, encoding BP100.2i and BP100.2mi, were purchased from GenScript (Piscataway NJ, USA) and included as well *BamHI* restriction sites, both at the 5’ and 3’ ends.

**Table 3:** Oligonucleotides used in this work

***Oligonucleotide code***	**Sequence**	**Length**
***recursive PCR***		
*PR1a.1_for*	5’ ATA Ggg atc cGA GGC CAC C*AT GGG CTT CGT CCT CTT CTC CCA ACT CCC ATC CTT CCT CCT CGT CTC CAC CCT CCT CCT GT* 3’	80
*BP100.1_ rev*	5’ GTA CTT GAG GAT CTT CTT GAA* GAG CTT CTT GGC GCG GCA GGA GTG GGA GAT CAC GAG GA A CAG GAG GAG GGT GGA* 3’	78
*BP100.2_ for*	5’ *GAA GCT* CTT CAA GAA GAT CCT CAA GTA CCT CGC CGG CCC AGC CAA GAA GCT CTT CAA GAA GAT CCT CAA GTA CCT CGC C 3’	82
*BP100.3KDEL_ rev*	5’ TAT Agg atc cAT TA**T CAG AGC TCG TC**C TTG AGG TAC TTG AGG ATC TTC TTG AAG AGC TTC TTG GCT GGG CCG GCG AGG TAC TTG AGG 3’	87
***Cloning steps***		
*PR1a_for*	5’ ATA Ggg atc cGA GGC CAC CAT 3’	21
*KDELbam_rev*	5’ TAT Agg atc cAT TAT CAG AGC TCG TC 3’	26
***Southern blot probe***		
*SouthUBI_for*	5’ ACA TGT GAT GTG GGT TTA CTG ATG 3’	24
*SouthBP_rev*	5’ GAG GTA CTT GAG GAT CTT CTT GAA G 3’	25
***qPCR***		
*bp100der_for*	5 ’ TCC TCG TGA TCT CCC ACT CCT G 3’	22
*bp100der_rev*	5’ CGG ATC CAT TAT CAG AGC TCG T 3’	22
*hgr_for*	5’ CGA AAT TGC CGT CAA CCA AGC 3’	21
*hgr_rev*	5’ CTG GAG CGA GGC GAT GTT C 3’	19

Underlined sequences indicate overlaps between two oligonucleotides used in recursive PCR, in italics is the *pr1a* signal sequence, the sequence encoding the KDEL retention signal is indicated in bold, and the *BamHI* and *KpnI* restriction sites introduced for cloning purposes are shown in lower case. Length of oligonucleotides is indicated in nt.

The newly synthesized *bp100.1*, *bp100.2*, *bp100.2i*, *bp100.3* and *bp100.2mi* genes were subcloned into the *BamHI* site of pAHC17 plasmid DNA [86], flanked by the promoter, first exon and first intron of the maize *ubiquitin* gene [87], previously shown to drive high expression levels of transgenes in rice [86,88], and the *Agrobacterium tumefaciens* nopaline synthase *nos* terminator sequences. After sequence verification by sequencing, the complete *bp100der* sequences (including promoter and terminator elements) were subcloned into the *KpnI* site of pCAMBIA1300, always in the opposite sense compared to the selection gene. The pCAMBIA derived binary vectors harboring *bp100der* cassettes were transferred into *Agrobacterium strain* EHA105 using the cold shock method [89]. DNA manipulations and transformation of the *Escherichia coli* strain XL1Blue with plasmid DNA were carried out using standard techniques [89].

### Production of transgenic rice plants

Transformation was carried out using the Mediterranean elite *japonica* rice (*Oryza sativa L*.) cultivar Senia. Transgenic lines expressing one of the *bp100der* genes were produced by *Agrobacterium* mediated transformation of embryonic callus derived from mature embryos as described by [90]. The control plasmid was pCAMBIA 1300 (with the resistance to hygromycin selection gene). T0 plants were grown to maturity and a selection of events was further cultured in standard greenhouse conditions to obtain homozygous transgenic lines in the T2 generation.

### Nucleic acid extraction

Genomic DNA was extracted from 1 g plant material using a CTAB based method [91]. Total RNA was extracted from 400 mg plant material, using a protocol based on the Trizol reagent (Invitrogen Life Technologies, Carlsbad, CA, USA), and purified using the Qiagen RNeasy MiniElute Cleanup kit (Qiagen, Hilden, Germany) according to the manufacturer’s instructions. DNA and RNA concentration and quality were systematically checked by UV absorption at 260 and 280 nm using a NanoDrop ND1000 spectrophotometer (Nanodrop technologies, Wilmington, DE, USA). All samples had appropriate values (mean and standard deviation [SD], 1.94 ± 0.15 and 2.08 ± 0.02 for DNA and RNA extracts, respectively).

### Southern blot

Genomic DNA (25 μg) was digested with *Hind III* or *Eco RI*, electrophoresed on 0.8% agarose gels, transferred to nylon membranes (Hybond-N, GE Healthcare Life Sciences*,* UK) and hybridized with a thermostable alkaline phosphatase labeled probe. The *bp100der* sequences were detected by chemiluminescence using the CDP-Star™ reagent (GE Healthcare Life Sciences*,* UK). The *bp100der* probe was obtained from a 406 bp PCR product encompassing part of the *ubi* first intron, the *pr1a* signal sequence and the first *bp134* element (PCR amplified with primers *SouthUBI_for* and *SouthBP_rev*, Table 3 and Figure 1) using the AlkPhos Direct Labeling and Detection System (GE Healthcare Life Sciences*,* UK). Hybridization and washes were carried out at 65°C according to the manufacturer’s instructions.

### Reverse transcription and PCR analysis

Transgene expression was assayed by reverse transcription coupled to real-time polymerase chain reaction (RT-qPCR). Reverse transcription was performed on 2,000 ng total RNA, previously treated with Turbo DNase (Ambion, Austin, TX, USA) using 50U of MultiScribe Reverse Transcriptase (Applied Biosystems, Foster City, CA, USA) and random hexamer primers (High-Capacity cDNA Reverse Transcription Kit, Applied Biosystems, Foster City, CA, USA) according to the manufacturer’s protocol. For each sample, cDNA was prepared at least in duplicate. The absence of remaining DNA targets was demonstrated by qPCR analyses (see below) of DNase-treated RNA samples.

qPCR assays targeting all five *bp100der* sequences and *hptII* were developed based on SYBR-Green technology. A single *bp100der* RT-qPCR assay was designed and optimized to detect and quantify *bp100.1*, *bp100.2*, *bp100.2i*, *bp100.3* and *bp100.2mi* based on two primers targeting conserved sites at the 5’ and 3’ ends of the coding sequences. PCR primers were designed using the Beacon Designer 7.0 software (Premier Biosoft International, Palo Alto, CA, USA). The qPCR assays were in a 20 μl volume containing 1X SYBR Green PCR Master Mix (Applied Biosystems, Foster City, CA, USA), 100 nM of primers (except for *hgr_for* and *hgr_rev*, 300nM and *bp100der_rev*, 50 nM) and 1 μl cDNA. Reaction conditions were: (1) initial denaturation (10 min at 95°C); (2) amplification and quantification (50 repeats of 15 s at 95°C and 1 min at 60°C) and (3) melting curve program (60-95°C with a heating rate of 0.5°C/s). Melting curve analyses produced single peaks, with no primer-dimer peaks or artifacts, indicating the reactions were specific. All oligonucleotides (Table 3) were purchased from MWG Biotech AG (Germany). Reactions were run on a 7500 Fast Real-Time PCR System (Applied Biosystems, Foster City, CA, USA) in triplicate. All reactions had linearity coefficient (R^2^) and efficiency values [E = 10^[−1/slope]^, [92]] above 0.99 and 0.95, respectively (for *bp100der* qPCR these values were assessed for each target).

The 18 S ribosomal RNA, β-actin and elongation factor (EF1α) housekeeping genes were analyzed as previously described [78]. Their suitability as internal standards was assessed in our samples through the geNORM v3.4 statistical algorithm, and β-actin had M values below 0.5 in all cases.

### Pathogen infection assays and oxidative stress assay

A total of ten homozygous plants of each GM event (3 S-bp100.1, 2 S-bp100.2i and 2 S-bp100.2mi lines), plus two control lines (i.e. untransformed Senia and S-hgr, transformed with the empty plasmid) were grown in the greenhouse to the vegetative three-leaf stage. The second leaves were detached and 50 mM H_2_O_2_ applied for 8 h, with subsequent in situ detection of O_2_^-^radicals in plant tissues by overnight staining with nitro blue tetrazolium (NBT). Leaves were visually inspected and the percentages of stained area were calculated using the APS assess v2.0 software tool.

A total of 15 homozygous seeds of each GM event, plus Senia and S-hgr lines, were surface sterilized and germinated in sterile water in 24-well culture chambers. They were incubated in a culture chamber at 28 ± 1°C with a photoperiod of 16 h light / 8 h dark under fluorescent Sylvania Cool White lamps. After overnight pre-germination they were inoculated with increasing concentrations of the bacterial plant pathogen *D. chrysanthemi *(10^2^, 10^3^, 10^4^, 10^5^ and 10^6^ for Senia; 10^5^ for all GM events) and allowed to continue germination for seven days. The development of *D. chrysanthemi*-infected seedlings from wild-type and transgenic plants was determined using a semi-quantitative scale (Figure 5A). Control seeds incubated in water confirmed germination of over 98% seeds, with no statistical difference among different events (one-way ANOVA *p* = 0.617). Uninfected seedlings from all analyzed events consistently had maximum development values.

For *F. verticillioides *(anamorph stage of *Gibberella fujikuroi*, mating population A) assays, nine seeds of each GM event, plus Senia and S-hgr lines, were surface sterilized and incubated for 30 min in the presence of a suspension of *F. verticillioides *conidia (10^5^ spores/ml) under agitation. They were allowed to germinate for seven days in sterile MS medium (Murashige and Skoog, 1962) supplemented with 0.7% agar in plastic tins under the same conditions as for *D.chrysanthemi*assays. Seedling development was estimated through a semi-quantitative index that considered shoot height (with values in the 0 to 1 range) and root development (number and length of crown roots and length of primary root, with values of 0 to 0.75 and 0 to 0.25, respectively). As uninfected control seeds consistently germinated and seedlings developed as expected they were given the highest possible values in this assay (i.e. 1, 0.75 and 0.25, respectively). Shoot height and primary root length were measured and values were assigned in the 0 to 1 and 0 to 0.25 ranges, respectively, in comparison to control seedlings. The number and length of crown roots were estimated by comparison with crown roots in control seedlings, and values were in the 0 to 0.5 and 0 to 0.25 ranges, respectively. The sum of the values for all seedlings was used to calculate the value of the seedling development index for each GM event. A global value of 0 indicated no germination and compact mycelium over the seed, while a value of 2 represented standard growth, the same as uninfected seeds.

### Agronomic characterization of GM plants

Transgenic and control lines were compared in terms of agronomic parameters. For S-bp100.2i and S-bp100.2mi lines (i.e. those expressing *bp100.2i* and *bp100.2mi*), T3 homozygous plants from all the independent events obtained were assayed. The assay was carried out in the quarantine greenhouse from 29 April to 7 October 2010, i.e. during the conventional rice growing season in the region. For each line, a total of 45 seeds were sown and 81 to 91% germinated. At the vegetative three-leaf stage a total of 30 plants per line (three replicates of 10 plants each) were transferred to pots and grown in the greenhouse under standard conditions. Plant growth was monitored every two weeks in terms of plant height, number of tillers, chlorophyll contents (spat) and flowering (a total of 5 observations, the last on 08/25/2010). To estimate yield related traits, the number of panicles per plant, number of grains per panicle and the weight per 100 grains were measured on harvesting. Plant yield was calculated as the product of number of panicles, number of grains per panicle and grain weight, and corresponds to the weight of all panicles in a plant.

### Bioinformatics

RT-qPCR data were normalized with the housekeeping gene and statistically analyzed using the SPSS software v.15.0 for Windows or the Genex software v.5.1.1.2 (MultiDAnalyses).

For pathogen resistance and oxidative stress tolerance assays, one-way ANOVA analyses were performed. The analytical results (i.e. percentages of NBT stained area or seedling development values in the presence of *D. chrysanthemi* or *F. verticillioides*) were used as the dependent variables, and the transgene (*bp100.2i*, *bp100.1*, *bp100.2mi*, *nptII* or none) as factor. Statistical analyses were performed jointly comparing the different events carrying the same *bp100der* to Senia.

## Competing interests

The authors declare that they have no competing interests.

## Authors’ contributions

MP, AN and EM conceived and designed the study. MM carried out most experiments and participated in the analysis of the data. MP supervised the study and wrote the paper. AN participated in the production of transgenic plants,microscopy, discussion of all experiments and preparation of the manuscript. NC helped in qRT-PCR assays. EB performed the in vitro assessment of the properties of BP100 derivatives. JM performed the agronomic characterization of GM plants. LM carried out pathogen resistance assays. EM participated in the design of BP100 derivatives and helped to draft the manuscript. All authors read and approved the final manuscript.

## Supplementary Material

Additional file 1 **Expression levels of *hptII *and the corresponding *bp100der *transgenes in three randomly chosen transgenic calluses per construct, as assessed by RT-qPCR.** Transgene mRNA copy numbers were normalized with actin values (GeNorm M values below 0.5). Means and SD of the three independent events are shown. No statistical differences were found. Click here for file

Additional file 2 **Transgene DNA copy numbers of S-bp100der plants.** (A) Southern blot analysis of transgenic lines. Genomic DNA was digested with the restriction enzymes EcoRI or Hind III and subjected to electrophoresis through a 0.8% agarose gel. DNAs were transferred to nylon membranes and hybridized with a thermostable alkaline phosphatase labelled probe. The migration positions and sizes of markers are indicated in base pairs on the left (MW). (B) Determination of transgene copy number by qPCR. Means of six experimental replicates are shown. RSD values were consistently below 2.5%. Transgene DNA copy numbers were normalized with actin values. Click here for file

Additional file 3 **Homozygous T2 rice lines obtained in this work and transgene mRNA expression values (relative to actin) in leaves of in vitro grown homozygous T3 plants, as assessed by RT-qPCR.** Mean and SD values corresponding to each particular GM event are shown. Three biological replicates per GM event were analyzed, each with leaves of 10 plants at the two-leaf stage. Click here for file

Additional file 4 Examples of Senia and S-bp100.2i plants at maturity. Click here for file

Additional file 5 **DNA sequences encoding the BP100 derivatives designed in this work.** The sequence encoding the Pr1a signal peptide is indicated in italics. The start and stop codons are underlined. Click here for file
