# Supplementary material for: Constitutive expression of transgenes encoding derivatives of the synthetic antimicrobial peptide BP100: impact on rice host plant fitness
Source: BMC Plant Biol. 2012 Sep 4;12:159. doi: 10.1186/1471-2229-12-159 (PMC3514116; doi:10.1186/1471-2229-12-159)
Supplement: Additional file 1 — Expression levels of hptII and the corresponding bp100der transgenes in three randomly chosen transgenic calluses per construct, as assessed by RT-qPCR. Transgene mRNA copy numbers were normalized with actin values (GeNorm M values below 0.5). Means and SD of the three independent events are shown. No statistical differences were found. [file 1471-2229-12-159-S5.docx]

**Additional File 1**
