# Supplementary material for: Constitutive expression of transgenes encoding derivatives of the synthetic antimicrobial peptide BP100: impact on rice host plant fitness
Source: BMC Plant Biol. 2012 Sep 4;12:159. doi: 10.1186/1471-2229-12-159 (PMC3514116; doi:10.1186/1471-2229-12-159)
Supplement: Additional file 2 — Transgene DNA copy numbers of S-bp100der plants. (A) Southern blot analysis of transgenic lines. Genomic DNA was digested with the restriction enzymes EcoRI or Hind III and subjected to electrophoresis through a 0.8% agarose gel. DNAs were transferred to nylon membranes and hybridized with a thermostable alkaline phosphatase labelled probe. The migration positions and sizes of markers are indicated in base pairs on the left (MW). (B) Determination of transgene copy number by qPCR. Means of six experimental replicates are shown. RSD values were consistently below 2.5%. Transgene DNA copy numbers were normalized with actin values. [file 1471-2229-12-159-S1.docx]

**Additional File 2**
