# Supplementary material for: Constitutive expression of transgenes encoding derivatives of the synthetic antimicrobial peptide BP100: impact on rice host plant fitness
Source: BMC Plant Biol. 2012 Sep 4;12:159. doi: 10.1186/1471-2229-12-159 (PMC3514116; doi:10.1186/1471-2229-12-159)
Supplement: Additional file 3 — Homozygous T2 rice lines obtained in this work and transgene mRNA expression values (relative to actin) in leaves of in vitro grown homozygous T3 plants, as assessed by RT-qPCR. Mean and SD values corresponding to each particular GM event are shown. Three biological replicates per GM event were analyzed, each with leaves of 10 plants at the two-leaf stage. [file 1471-2229-12-159-S2.docx]

**Additional File 3**
