# Supplementary material for: Constitutive expression of transgenes encoding derivatives of the synthetic antimicrobial peptide BP100: impact on rice host plant fitness
Source: BMC Plant Biol. 2012 Sep 4;12:159. doi: 10.1186/1471-2229-12-159 (PMC3514116; doi:10.1186/1471-2229-12-159)
Supplement: Additional file 4 — Examples of Senia and S-bp100.2i plants at maturity. [file 1471-2229-12-159-S3.docx]

**Additional File 4**
