# Supplementary figures and images for: Constitutive expression of transgenes encoding derivatives of the synthetic antimicrobial peptide BP100: impact on rice host plant fitness
Source: BMC Plant Biol. 2012 Sep 4;12:159. doi: 10.1186/1471-2229-12-159 (PMC3514116; doi:10.1186/1471-2229-12-159)

**Additional File 5**


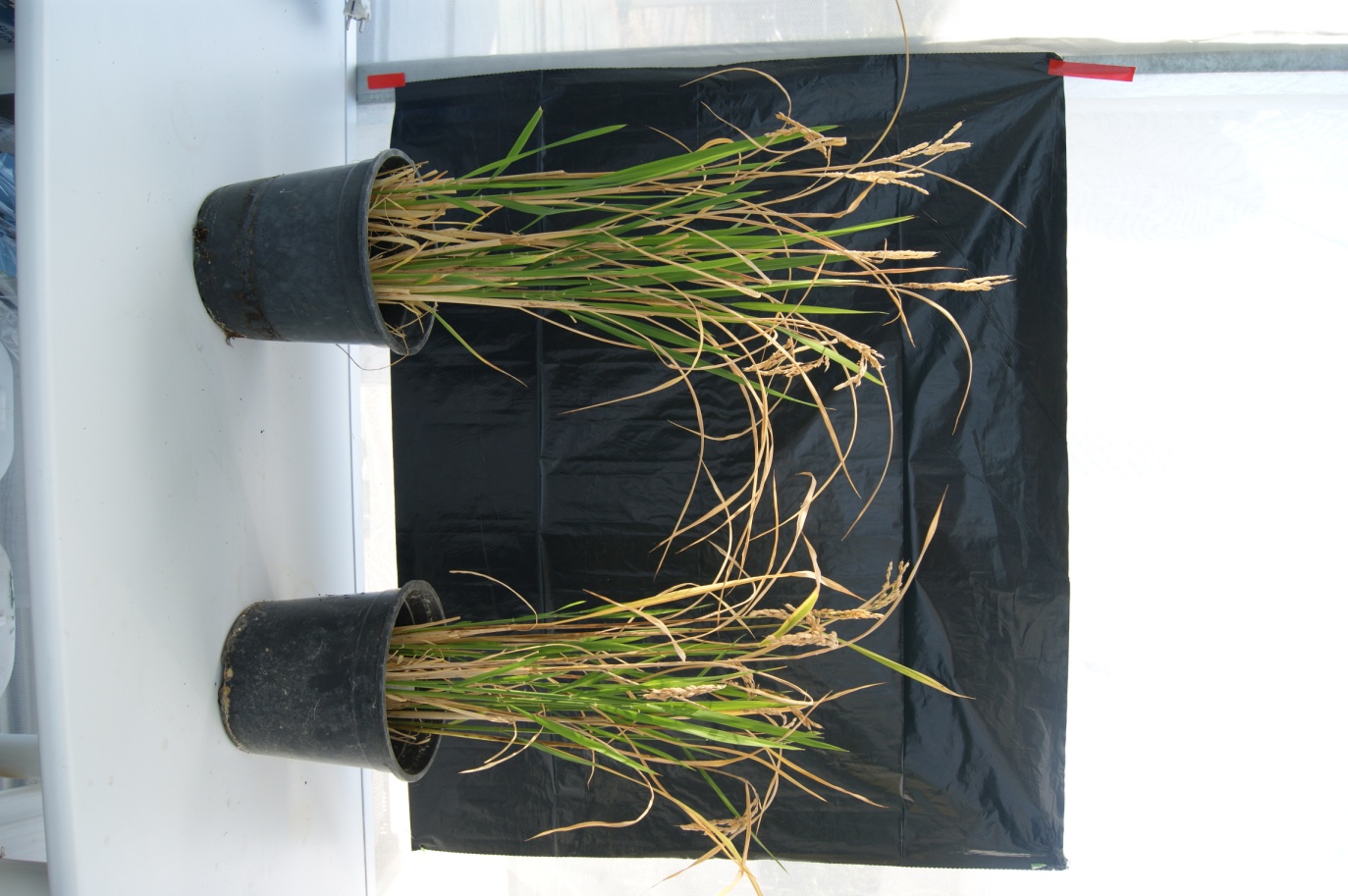


Senia

S-bp100.2i

Supplement: Additional file 5 — DNA sequences encoding the BP100 derivatives designed in this work. The sequence encoding the Pr1a signal peptide is indicated in italics. The start and stop codons are underlined. [file 1471-2229-12-159-S4.docx]
